# Supplementary material for: Punishment and inspection for governing the commons in a feedback-evolving game
Source: PLoS Comput Biol. 2018 Jul 20;14(7):e1006347. doi: 10.1371/journal.pcbi.1006347 (PMC6070290; doi:10.1371/journal.pcbi.1006347)
Supplement: S1 Text — (PDF) [file pcbi.1006347.s001.pdf]

SI text (Supporting Information): Punishment and inspection for governing the commons in a feedback-evolving game

Xiaojie Chen[1*] and Attila Szolnoki[2†]

1 School of Mathematical Sciences, University of Electronic Science and Technology of China, Chengdu 611731, China
2 Institute of Technical Physics and Materials Science, Centre for Energy Research, Hungarian Academy of Sciences, P.O. Box 49, H-1525 Budapest, Hungary

* xiaojiechen@uestc.edu.cn
† szolnoki@mfa.kfki.hu

In this text, we provide the theoretical analysis of the equilibrium points in the following special cases.

1 For $0 < r = e_c < e_d$

In this case, we have $r = \frac{b_m N}{R_m}$. Accordingly, the equation system becomes

$$\begin{cases} \dot{x} = x(1-x)(p\beta - \dfrac{y}{R_m} b_m \alpha) \\ \dot{y} = -\alpha r y + \alpha r x y - \dfrac{r y^2}{R_m}. \end{cases}$$

Then the system has two fixed points in the parameter space, which are $[0,0]$ and $[1,0]$, respectively. Here the largest eigenvalue of $J(0,0)$ is positive, thus the fixed point $[0,0]$ is unstable.

For the fixed point $[1,0]$, we have

$$J(1,0) = \begin{bmatrix} -p\beta & 0 \\ 0 & 0 \end{bmatrix}.$$

Then we cannot determine the stability of this fixed point based only on the eigenvalues of the Jacobian. Instead, we study its stability by further using the center manifold theorem [1]. To do that, we take $x - 1 = z$, then the equation system becomes

$$\begin{cases} \dot{y} = \alpha r y z - \dfrac{r y^2}{R_m} \\ \dot{z} = -p\beta z + \dfrac{\alpha b_m y z}{R_m} - p\beta z^2 + \dfrac{\alpha b_m y z^2}{R_m}. \end{cases}$$

Using the center manifold theorem, we have that $z = h(y)$ is a center manifold for the above system. We start to try $h(y) = O(|y|^2)$, which yields the reduced system

$$\dot{y} = -\frac{r y^2}{R_m} + O(|y|^3).$$

Since the fraction $\frac{r}{R_m}$ is nonzero, the fixed point $y = 0$ of the reduced system is unstable. Consequently, the fixed point $[1,0]$ is unstable as well [1].

2 For $0 < e_c < r < e_d$ and $\alpha b_m(1-\frac{e_c}{r}) = p\beta$

In this case, we have $1+\frac{1}{\alpha}-\frac{R_m r}{\alpha b_m N}+\frac{p\beta R_m r}{\alpha^2 b_m^2 N}<1$ and $R_m-\frac{Nb_m}{r}=\frac{p\beta R_m}{\alpha b_m}$. As a result, there are three fixed points in the parameter space, which are $[0,0]$, $[1,0]$, and $[1, R_m-\frac{Nb_m}{r}]$, respectively.

The fixed points $[0,0]$ and $[1,0]$ are both unstable since the largest eigenvalues of $J(0,0)$ and $J(1,0)$ are both positive.

For the fixed point $[1, R_m-\frac{Nb_m}{r}]$, we have

$$J(1, R_m-\tfrac{Nb_m}{r}) = \begin{bmatrix} 0 & 0 \\ Np\beta & \frac{Nb_m - R_m r}{R_m} \end{bmatrix},$$

in which one eigenvalue is zero and the other eigenvalue is negative. Accordingly, we study its stability by further using the center manifold theorem [1]. To do that, we take $x-1=z$ and $w=y-(R_m-\frac{Nb_m}{r})$, then the equation system becomes

$$\begin{cases} \dot{z} = -z(z+1)[p\beta - \dfrac{\alpha b_m(rw+R_m r-Nb_m)}{R_m r}] \\ \dot{w} = \dfrac{[r(R_m+w)-Nb_m](\alpha b_m N z - rw)}{R_m r}. \end{cases}$$

We further let M be a matrix whose columns are the eigenvectors of $J(1, R_m-\frac{Nb_m}{r})$, which can be written as

$$M = \begin{bmatrix} \frac{R_m r - Nb_m}{Np\beta R_m} & 0 \\ 1 & 1 \end{bmatrix}.$$

Then we have

$$M^{-1}J(1, R_m-\tfrac{Nb_m}{r})M = \begin{bmatrix} 0 & 0 \\ 0 & \frac{Nb_m - R_m r}{R_m r} \end{bmatrix}.$$

We further take $[u \quad v]^T = M^{-1}[z \quad w]^T$, and thus we have $u=\frac{Np\beta R_m}{R_m r - Nb_m}z$ and $v=w-u$. It leads to

$$\dot{u} = \frac{\alpha b_m}{R_m}uv + \frac{\alpha b_m}{R_m}u^2 + \frac{ru^2v}{R_m N} + \frac{ru^3}{R_m N}.$$

Using the center manifold theorem, we have that $v=h(u)$ is a center manifold. We start to try $h(u)=O(|u|^2)$, which yields the reduced system

$$\dot{u} = \frac{\alpha b_m}{R_m}u^2 + \frac{u^3}{R_m N} + O(|u|^4).$$

Since $\frac{\alpha b_m}{R_m} \neq 0$, the fixed point $u=0$ of the reduced system is unstable. Consequently, the fixed point $[1, R_m-\frac{Nb_m}{r}]$ of the original system is unstable [1].

3 For $0 < e_c < r = e_d$

In this case, we have $1+\frac{1}{\alpha}-\frac{R_m r}{\alpha b_m N}=0$. In dependence of the efficiency of inspection and punishment we can further distinguish three following subcases.

(1) For $b_m\alpha(1-\frac{e_c}{r}) < p\beta$

We then have $1+\frac{1}{\alpha}-\frac{R_m r}{\alpha b_m N}+\frac{p\beta R_m r}{\alpha^2 b_m^2 N}=\frac{p\beta R_m r}{\alpha^2 b_m^2 N}>1$. As a result, the system has three fixed points in the parameter space. They are $[0,0]$, $[1,0]$, and $[1, R_m-\frac{Nb_m}{r}]$,

respectively. According to the sign of the largest eigenvalues in the Jacobian matrices, $[0,0]$ and $[1,0]$ are unstable, while $[1, R_m - \frac{Nb_m}{r}]$ is a stable fixed point.

(2) For $b_m\alpha(1-\frac{e_c}{r}) > p\beta$

We then have $1+\frac{1}{\alpha}-\frac{R_m r}{\alpha b_m N}+\frac{p\beta R_m r}{\alpha^2 b_m^2 N}=\frac{p\beta R_m r}{\alpha^2 b_m^2 N}<1$. As a result, the system has four fixed points in the parameter space. They are $[0,0]$, $[1,0]$, $[1, R_m-\frac{Nb_m}{r}]$, and $[\frac{p\beta R_m r}{\alpha^2 b_m^2 N}, \frac{p\beta R_m}{\alpha b_m}]$, respectively. The first three fixed points are all unstable since the sign of the largest eigenvalues of the Jacobian matrices are all positive, while the last fixed point is stable because the term $\frac{p\beta R_m r}{\alpha b_m^2 N}-\frac{R_m r}{N b_m}+1$ is negative.

(3) For $b_m\alpha(1-\frac{e_c}{r}) = p\beta$

We then have $1+\frac{1}{\alpha}-\frac{R_m r}{\alpha b_m N}+\frac{p\beta R_m r}{\alpha^2 b_m^2 N}=\frac{p\beta R_m r}{\alpha^2 b_m^2 N}=1$ and $R_m-\frac{Nb_m}{r}=\frac{p\beta R_m}{\alpha b_m}$. As a result, the system has three fixed points in the parameter space. They are $[0,0]$, $[1,0]$, and $[1, \frac{p\beta R_m}{\alpha b_m}]$, respectively.

The fixed points $[0,0]$ and $[1,0]$ are both unstable since the largest eigenvalues of $J(0,0)$ and $J(1,0)$ are both positive.

For the fixed point $[1, \frac{p\beta R_m}{\alpha b_m}]$, we have

$$J(1, \tfrac{p\beta R_m}{\alpha b_m}) = \begin{bmatrix} 0 & 0 \\ Np\beta & -\frac{p\beta r}{\alpha b_m} \end{bmatrix},$$

in which one eigenvalue is zero and the other eigenvalue is negative. Accordingly, we study its stability by further using the center manifold theorem [1]. To do that, we take $x-1=z$ and $w=y-\frac{p\beta R_m}{\alpha b_m}$, then the equation system becomes

$$\begin{cases} \dot{z} = \dfrac{\alpha}{R_m} b_m z(z+1)w \\ \dot{w} = -\dfrac{r}{R_m}(w+\dfrac{p\beta R_m}{\alpha b_m})^2 + \dfrac{N\alpha b_m}{R_m}(z+1)(w+\dfrac{p\beta R_m}{\alpha b_m}). \end{cases}$$

We further let M be a matrix whose columns are the eigenvectors of $J(1, \frac{p\beta R_m}{\alpha b_m})$, which can be written as

$$M = \begin{bmatrix} \frac{r}{\alpha b_m N} & 0 \\ 1 & 1 \end{bmatrix}.$$

Then we have

$$M^{-1} J(1, \tfrac{p\beta r}{\alpha b_m}) M = \begin{bmatrix} 0 & 0 \\ 0 & -\frac{p\beta r}{\alpha b_m} \end{bmatrix}.$$

We further take $[u \quad v]^T = M^{-1}[z \quad w]^T$, and thus we have $u=\frac{\alpha b_m N}{r}z$ and $v=w-u$. It leads to

$$\dot{u} = \frac{\alpha b_m}{R_m}uv + \frac{\alpha b_m}{R_m}u^2 + \frac{ru^2 v}{R_m N} + \frac{ru^3}{R_m N}.$$

Using the center manifold theorem, we have that $v=h(u)$ is a center manifold. We start to try $h(u)=O(|u|^2)$, which yields the reduced system

$$\dot{u} = \frac{\alpha b_m}{R_m}u^2 + \frac{u^3}{R_m N} + O(|u|^4).$$

Since $\frac{\alpha b_m}{R_m} \neq 0$, the fixed point $u=0$ of the reduced system is unstable. Consequently, the fixed point $[1, \frac{p\beta r}{\alpha b_m}]$ of the original system is unstable [1].

4 For $0 < e_c < e_d < r$ and $\alpha b_m(1 - \frac{e_c}{r}) = p\beta$

In this case, we have $1 + \frac{1}{\alpha} - \frac{R_m r}{\alpha b_m N} + \frac{p\beta R_m r}{\alpha^2 b_m^2 N} = 1$ and $R_m - \frac{N b_m}{r} = \frac{p\beta R_m}{\alpha b_m}$. As a result, there are four fixed points, which are $[0, 0]$, $[1, 0]$, $[0, R_m - \frac{N b_m(1+\alpha)}{r}]$, and $[1, R_m - \frac{N b_m}{r}]$, respectively. The first three fixed points are all unstable since the sign of the largest eigenvalues of the Jacobian matrices are all positive. In addition, based on the theoretical analysis in Sec. 2 of this text, we can conclude that the fixed point $[1, R_m - \frac{N b_m}{r}]$ is also unstable.

5 For $0 < e_c < e_d < r$ and $\alpha b_m(1 - \frac{e_d}{r}) = p\beta$

In this case, we have $1 + \frac{1}{\alpha} - \frac{R_m r}{\alpha b_m N} + \frac{p\beta R_m r}{\alpha^2 b_m^2 N} = 0$ and $R_m - \frac{N b_m(1+\alpha)}{r} = \frac{p\beta R_m}{\alpha b_m}$. As a result, the system has four fixed points in the parameter space. They are $[0, 0]$, $[1, 0]$, $[1, R_m - \frac{N b_m}{r}]$, and $[0, \frac{p\beta R_m}{\alpha b_m}]$, respectively.

The fixed points $[0, 0]$, $[1, 0]$, and $[1, R_m - \frac{N b_m}{r}]$ are all unstable since the largest eigenvalues of $J(0, 0)$, $J(1, 0)$, and $J(1, R_m - \frac{N b_m}{r})$ are all positive.

For the fixed point $[0, \frac{p\beta R_m}{\alpha b_m}]$, we have

$$J(0, \tfrac{p\beta R_m}{\alpha b_m}) = \begin{bmatrix} 0 & 0 \\ Np\beta & -\frac{p\beta r}{\alpha b_m} \end{bmatrix},$$

in which one eigenvalue is zero and the other eigenvalue is negative. Accordingly, we study its stability by using again the center manifold theorem [1]. To do that, we take $x = z$ and $w = y - \frac{p\beta R_m}{\alpha b_m}$, then the equation system becomes

$$\begin{cases} \dot{z} = \dfrac{\alpha b_m}{R_m} z(z-1)w \\ \dot{w} = -N b_m(1+\alpha-\alpha z)(\dfrac{w}{R_m} + \dfrac{p\beta}{\alpha b_m}) + r(w + \dfrac{p\beta R_m}{\alpha b_m})(1 - \dfrac{w}{R_m} - \dfrac{p\beta}{\alpha b_m}). \end{cases}$$

We further let M be a matrix whose columns are the eigenvectors of $J(0, \frac{p\beta R_m}{\alpha b_m})$, which can be written as

$$M = \begin{bmatrix} \frac{r}{\alpha b_m N} & 0 \\ 1 & 1 \end{bmatrix}.$$

Then we have

$$M^{-1} J(0, \tfrac{p\beta r}{\alpha b_m}) M = \begin{bmatrix} 0 & 0 \\ 0 & -\frac{p\beta r}{\alpha b_m} \end{bmatrix}.$$

We further take $[u \quad v]^T = M^{-1}[z \quad w]^T$, and thus we have $u = \frac{\alpha b_m N}{r} z$ and $v = w - u$. It leads to

$$\dot{u} = -\frac{r}{R_m N} uv - \frac{r}{R_m N} u^2 + \frac{r^2 u^2 v}{R_m \alpha b_m N^2} + \frac{r^2 u^3}{R_m \alpha b_m N^2}.$$

Using the center manifold theorem, we have that $v = h(u)$ is a center manifold. We start to try $h(u) = O(|u|^2)$, which yields the reduced system

$$\dot{u} = -\frac{r}{R_m N} u^2 + \frac{r^2 u^3}{R_m \alpha b_m N^2} + O(|u|^4).$$

Since the fraction $\frac{r}{R_m N}$ is nonzero, the fixed point $u = 0$ of the reduced system is unstable. Consequently, the fixed point $[0, \frac{p\beta R_m}{\alpha b_m}]$ of the original system is unstable [1].

References

1. Khalil HK. Nonlinear Systems. Prentice Hall, NJ 1996.
